# Virulent Properties of Russian Bovine Viral Diarrhea Virus Strains in Experimentally Infected Calves

**DOI:** 10.1155/2016/7034509

**Published:** 2016-04-14

**Authors:** Alexander G. Glotov, Tatyana I. Glotova, Svetlana V. Koteneva, Olga V. Semenova, Alexander A. Sergeev, Ksenya A. Titova, Anastasia A. Morozova, Artemiy A. Sergeev

**Affiliations:** ^1^Institute of Experimental Veterinary Science of Siberia and the Far East, Krasnoobsk, Novosibirsk Region 630501, Russia; ^2^State Research Center of Virology and Biotechnology Vector, Koltsovo, Novosibirsk Region 630559, Russia

## Abstract

The results of experimental study of three noncytopathic and two cytopathic bovine viral diarrhea virus (BVDV) strains isolated from cattle in the Siberian region and belonging to the type 1 (subtypes 1a, 1b, and 1d) have been presented. All investigated strains caused the development of infectious process in the seronegative 4–6-month-old calves after aerosol challenge with the dose of 6 log_10_ TCID_50_. The greatest virulence had noncytopathic strain and cytopathic strain related to the subtypes 1d and 1b, respectively. All strains in infected calves caused some signs of moderate acute respiratory disease and diarrhea: depression 3–5 days postinfection (p.i.), refusal to food, severe hyperthermia to 41.9°С, serous exudate discharges from the nasal cavity and eyes, transient diarrhea with blood, leukopenia (up to 2700 cells/mm^3^), and macroscopic changes in the respiratory organs and intestine. The infected animals recovered from 12 to 15 days p.i. and in 90% cases formed humoral immune response 25 days p.i. (antibody titers to BVDV: 1 : 4–1 : 16). Our results confirmed the presence of virulent BVDV1 strains and showed the need for researches on the molecular epidemiology of the disease, development of more effective diagnostic systems, and optimization of control programs with use of vaccines.

## 1. Introduction

The Russian livestock industry, notably the dairy cattle industry, is currently facing serious changes. The number of large dairy farms, counting from 800 to 1,500 dairy cows with an average annual milk production of about 12,000 kg, is increasing significantly. Highly productive breeding animals are imported to some Russian regions from around the world: USA, Canada, Holland, Denmark, France, Germany, Austria, Hungary, and Slovenia. Extensive movement of animals from multiple sources, the hallmark of large livestock importation programs such as this, bears the risk of the high probability for introduction of transmissible infectious diseases including bovine viral diarrhea virus (BVDV) infections, causing diseases in infected cattle that are economically important. Consequently, the importance of launching state-of-the-art BVDV research activities based on contemporary technology to facilitate implementation of effective practices to control and prevent the negative effects of BVDV infection in Russian cattle is clearly evident for the country and particularly for Siberia. Adult cattle seropositivity, indicating an early-borne infection, varies from 75 to 95% in six regions of Siberia (Glotov, unpublished data). In this situation, information on the circulation of the pathogen among susceptible animals is needed. This data may serve as a foundation for designing and evaluating diagnostic tools and for choosing more effective vaccines; therefore, it may potentially be a contributing factor in controlling BVDV infection. There are no data on genotyping BVDV and the study of virulent properties of this virus in the naturally susceptible animals in the available Russian literature.

Bovine viral diarrhea virus is widespread throughout the world and causes significant economic damage to dairy and beef cattle [1, 2]. Two biotypes of the pathogen of the disease (bovine viral diarrhea virus, BVDV), cytopathic and (more frequent) noncytopathic [2–4], as well as two types, 1 and 2 [5], have been identified. Now it allocates at least 16 subtypes (1a–1o) of BVDV1 [6, 7] and at least five subtypes (2a–2e) of the BVDV2 [8, 9]. Type 2 strains are less common than type 1 strains but are more virulent [10, 11].

Bovine viral diarrhea is often subclinical [1, 2]. Noncytopathic biotype plays the greatest role in the epidemiology and is the most virulent as it causes transplacental infection, leading to reproductive disorders in cows and to persistent infection of the fetus, as well as immunosuppression in acute forms of postnatal infection [4]. Mechanism of the immunosuppressive effect of the virus includes leukopenia, decreased lymphocyte proliferation, depletion of lymphoid tissues, decreased chemotaxis and phagocytic activity of macrophages, increased production of prostaglandin E2, and violation generation of proinflammatory cytokines [12–14]. The immunosuppression is a transient (2-3 weeks) or long-term nature in persistently infected animals. It is believed that the virus is not a direct respiratory pathogen, but its ability to cause immunosuppression significantly increases the risk of respiratory disease in calves, related in particular to the multiplication of viruses of other nosological groups and bacteria of Pasteurellaceae family [15–17].

The allocation of the new cytopathic and noncytopathic BVDV strains instigates scientists around the world to study their virulent properties, including the ability to induce development of infection in the susceptible animals. In this regard, these studies have not been conducted with strains of the virus isolated in the Russian territory.

The aim of this study was to determine virulent properties of cytopathic and noncytopathic BVDV1 strains belonging to three different subtypes and isolated from cattle in the Siberian region in seronegative calves.

## 2. Materials and Methods

### 2.1. Virus

For experimental infection five BVDV1 strains of three subtypes which have been isolated from cattle in the Siberian region were used [10, 18]. Moreover, three strains taken in the study were noncytopathic (T2, Bor, and Bison) and belonged to different subtypes, 1a, 1b, and 1d, respectively, while the remaining two were cytopathic (B1 and T1) of the same subtype, 1b. Virus-containing suspensions that were prepared by culturing BVDV1 strains in primary calf testis cells cultures had concentration from 6.0 to 6.5 log_10_ TCID_50_/0.1 cm^3^ (50% tissue culture infective dose per 0.1 cm^3^). This virus titration was performed traditionally by micromethod with using the same cell culture and 96-well flat-bottomed culture plates (TPP Techno Plastic Products AG, Switzerland). After 5–7 days of virus cultivation at 37°C we registered the presence of cytopathic effect in the wells of the cell culture infected with cytopathic strains and the presence of BVDV1 genetic material by polymerase chain reaction (PCR) in the wells of the cell culture infected with noncytopathic strains, expressing the titer values in TCID_50_/0.1 cm^3^. Virus-containing material was packaged in individual vials and stored at minus 80°C.

### 2.2. Cell Cultures

Testes were obtained from calves that did not contain the virus in blood and antibodies to it that we diagnosed by PCR and serum neutralization test before the study. This cell culture was used for propagation and titration of virus strains. Continuous Madin-Darby bovine kidney (MDBK) cell culture line was obtained from the Cell Culture Collection of State Research Center of Virology and Biotechnology Vector (SRC VB Vector), was free from BVDV1 and BVDV2 contaminations, according to PCR results, and was used exclusively for serum neutralization test to detect antibodies to the virus in experienced and control calves. The cell monolayers were grown in Eagle's minimum essential medium (EMEM) (OJSC BioloT, Russia) in the presence of 10% fetal bovine serum (HyClone, USA) supplemented with penicillin (100 IU/cm^3^) and streptomycin (100 *μ*g/cm^3^). This serum was free from “accidental” BVDV contamination and specific antibodies according to both the manufacturer guarantee (HyClone, USA) and additional carried out testing by PCR and serum neutralization test. The same medium supplemented with 2% fetal bovine serum, penicillin (100 IU/cm^3^), and streptomycin (100 *μ*g/cm^3^) was used as a supporting one for the virus cultivation.

### 2.3. Experimental Animals

The study involved the use of 21 Holstein Friesian black and white breed 4–6-month-old calves (body weight of about 150 kg), obtained from the farm free of bovine viral diarrhea and other viral diseases during the last 5 years. These animals were grown in individual cages by a method of moderately low temperatures and were not exposed to stress during transport and feeding. These animals did not contain BVDV in blood (absence of virus in the blood and its genetic material as well as antibodies thereto in blood serum). They were also seronegative for bovine herpesvirus 1 which excluded the presence of mixed infection. All calves were divided into 7 groups (five experimental and two control) for three animals in each. Handling of calves was conducted in accordance with the Animal Welfare Act as amended (7 United States Code, 2131-2156). Research and manipulations on animals were conducted with the approval of SRC VB Vector's Bioethics Committee # 1-01.2014 dated January 28, 2014.

### 2.4. Method and Dose Used for Calves Challenge

Experimental infection of calves was performed with virus-containing suspensions of a BVDV1 strains in a concentration of 6 log_10_ TCID_50_/0.1 cm^3^ by a pneumatic type BG-2 atomizer (SRC VB Vector, Russia) creating an aerosol with a mass median aerodynamic diameter of particles of 5 microns. With this method, the suspensions were injected in a volume of 1 cm^3^ to the nasal cavity of animals during inhalation acts performed by them. Thus each animal received a dose of 6 log_10_ TCID_50_ of one or the other virus strain. Control animals received in the same manner saline in a volume of 1 cm^3^. For all of the animals the observation was conducted within 25 days with daily thermometry by electronic thermometer “Thera-moval” (HARTMAN, Germany). The average value of the calf temperature before infection was 39.0 ± 0.5°C.

### 2.5. Preparation of Samples from the Experimental and Control Calves

10 days postinfection (p.i.) one calf from each test and control group was euthanized to study the macroscopic lesions in the internal organs and to identify the viral genetic material in them as compared with uninfected animals. For this purpose, samples of the nasal mucosa, trachea, nasopharynx, thymus, lung, and mesenteric lymph nodes, bronchial, tracheal, and bronchial exudates, lung, tonsil, spleen, small and large intestines, duodenum, ileum, and mesenteric lymph nodes were taken. To evaluate hematological parameters in experimental and control calves were bled one day before infection and daily within 25 days p.i. The sera obtained one day before infection and 25 days p.i. were used to determine seroconversion in these animals.

### 2.6. Assessment of Antibody Titers in Calves

Serum neutralization test was performed in accordance with the Office International des Epizooties (OIE) by micromethod in MDBK cell culture using cytopathic BVDV strain NADL (subtype 1a) as antigen [19].

### 2.7. PCR Analysis

The detection of BVDV RNA in the internal organs of infected animals was carried out by PCR using primers proposed by Ridpath et al. [20]. The primer set for the first round of PCR (for detection of BVDV1 and BVDV2) 5′UTR is as follows:

 left primer: 5′CAT GCC CAT AGT AGG AC 3′;
 right primer: 5′CCA TGT GCC ATG TAC AG 3′.
 To differentiate BVDV1 isolates the following primers were used:
 to identify subtype 1a:
 left primer: 5′TCG ACG CCT TRR CAT GAAGGT 3′;
 right primer: 5′CCA TGT GCC ATG TAC AG 3′;
 to identify subtype 1b:
 left primer: 5′TCG ACG CTTTGG AGGACAAGC 3′;
 right primer: 5′CCA TGT GCC ATG TAC AG 3′.
 Additionally to identify subtype 1d (E2 gene) of BVDV we used our own designed primers [10]:
 left primer: F-BV-2275 (AYT GGT GGC CWT ATG AGA C);
 right primer: R-BV-3434 (YAG GTC AAA CCA RTA TTG).

### 2.8. Hematological Investigation

Blood samples obtained from the control and experimental calves were examined by calculating the concentration of leukocytes in conventional manner [21]. A total of 10 cm^3^ of peripheral blood samples was collected in Vacutainer tubes containing ethylenediaminetetraacetic acid, EDTA (Beckton Dickinson, Franklin Lakes, NJ, USA). Total leukocytes were counted using a Vet Scan HM5 hematology system (Abaxis, Union, CA, USA).

### 2.9. Statistical Treatment of Results

Statistical treatment of results was carried out with standard methods [22] using the software package Statistica 6.0 (StatSoft Inc. 1984–2001) with assessment of significant differences for *p* ≤ 0.05 (*I*_95_).

## 3. Results

### 3.1. The Study of Virulent Properties of Noncytopathic Bovine Viral Diarrhea Virus Strains on Experimentally Infected Calves

In the first series of experiments to aerosol challenge nine calves were infected by three noncytopathic BVDV strains, relating to the same type but differing in subtypes (1a, 1b, and 1d). The results of these studies are presented in Table 1.

**Figure 1:**
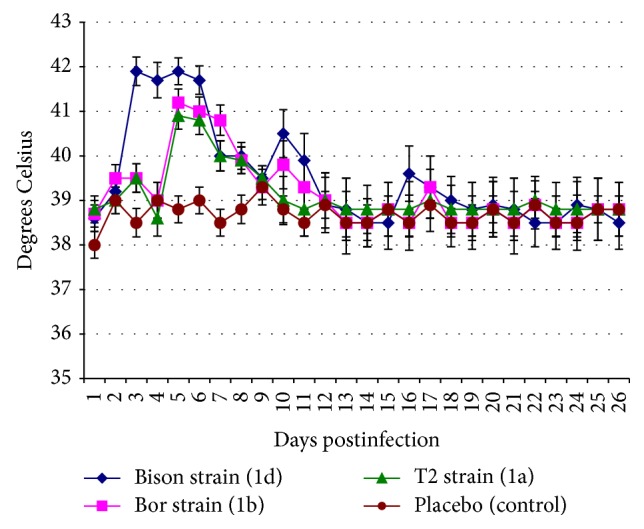
The data on the dynamics of rectal temperature in 4–6-month-old calves after aerosol challenge by bovine viral diarrhea virus different noncytopathic strains of type 1 with the dose of 6 log_10_ TCD_50_. The values are presented as *M* ± *I*_95_ (*M*, mean value; *I*_95_, 95% confidence interval) for *n* = 3, the number of animals per time point (1–10 days postinfection, p.i.); *n* = 2, the number of animals per time point (11–25 days p.i.); values obtained for the three strains 5–8 days p.i. are higher than in the control according to Mann-Whitney *U* test and Student's *t*-test at *p* ≤ 0.05; values obtained for Bison strain 3–6 days p.i. are higher than for T2 and Bor strains according to Mann-Whitney *U* test and Student's *t*-test at *p* ≤ 0.05; values obtained for Bison strain 10 and 11 days p.i. are higher than for T2 strains according to Mann-Whitney *U* test and Student's *t*-test at *p* ≤ 0.05.

**Figure 2:**
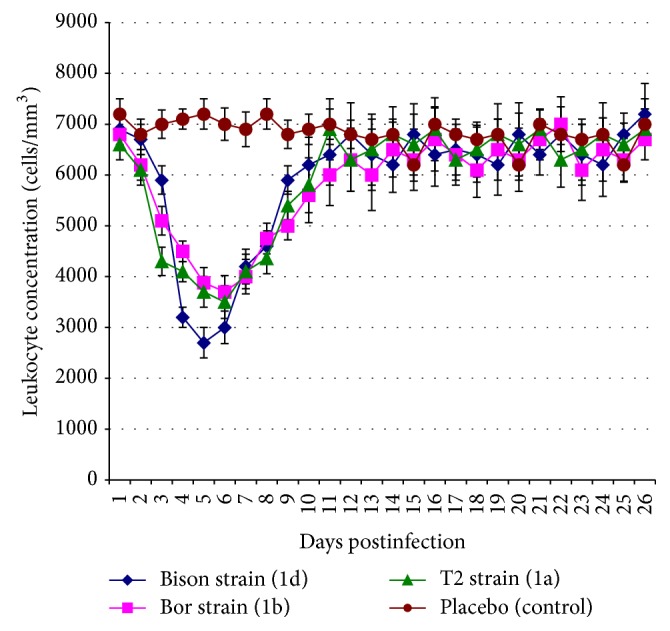
The data on the dynamics of leukocyte concentration in 4–6-month-old calves after aerosol challenge by bovine viral diarrhea virus different noncytopathic strains of type 1 with the dose of 6 log_10_ TCD_50_. The values are presented as *M* ± *I*_95_ (*M*, mean value; *I*_95_, 95% confidence interval) for *n* = 3, the number of animals per time point (1–10 days postinfection, p.i.); *n* = 2, the number of animals per time point (11–25 days p.i.); values obtained for the three strains 3–9 days p.i. are lower than in the control according to Mann-Whitney *U* test and Student's *t*-test at *p* ≤ 0.05; values obtained for Bison strain 4–6 days p.i. are less than for Bor and T2 strains according to Mann-Whitney *U* test and Student's *t*-test at *p* ≤ 0.05; values obtained for Bor strain 3 days p.i. are less than for T2 and Bison strains according to Mann-Whitney *U* test and Student's *t*-test at *p* ≤ 0.05.

It was shown that all BVDV1 strains (isolated from calf lungs, bull sperm, and heifer blood serum) after the same administered dose (6 log_10_ TCD_50_) induced the development of infectious process but its manifestation intensity varied. The most virulent properties had Bison strain, previously taken from calf lungs, which caused clinical signs of mild respiratory disease manifested in the form of depression 4-5 days p.i., refusal to food, expressed body hyperthermia (Figure 1), serous exudate discharges from the nasal cavity and eyes, and transient diarrhea with blood in all cases. Moreover, animals infected with this strain demonstrated significantly higher body temperature and lower blood leukocyte concentration (Figure 2) than calves after aerosol challenge with other noncytopathic BVDV1 strains. One experimental calf marked short-term enlargement of subscapular lymph node. Infected animals recovered from 13 to 15 days p.i. The autopsy of calf on 10th day p.i. showed the presence of serous fluid in the upper respiratory tract and hyperemia of the small intestine. Furthermore, there was wide spread of the BVDV1 genetic material to organs and tissues compared to the one that was observed in calves infected with other noncytopathic strains of the virus.

Aerosol administration of BVDV1 Bor strain to calves resulted in a slight depression, loss of appetite within 6–10 days p.i., pyrexia (Figure 1), and leukopenia (Figure 2). Recovery of the animals of this group was recorded from 12 to 14 days p.i. The autopsy of one of the calves on 10th day p.i. demonstrated a slight enlargement only in mesenteric lymph nodes and hyperemia of small intestine mucosa. BVDV1 T2 strain administration caused the signs of the disease in all infected animals similar to those seen in animals challenged with a Bor strain.

The 4–16-fold seroconversion to BVDV1 was detected in 5 of 6 experimental animals after 25 days p.i. which confirms development of acute viral infection in calves. The result of serological examination of one calf infected with BVDV1 T2 strain was negative. No changes in all the studied parameters for the entire period of observation in the control animals after challenge with aerosolized saline were registered.

### 3.2. Study of Virulent Properties of Cytopathic Bovine Viral Diarrhea Virus Strains on Experimentally Infected Calves

The aerosol challenge of calves was performed by two BVDV1 cytopathic strains both previously allocated from calf lungs and belonging to subtype 1b. The results of these studies are presented in Table 2.

**Figure 3:**
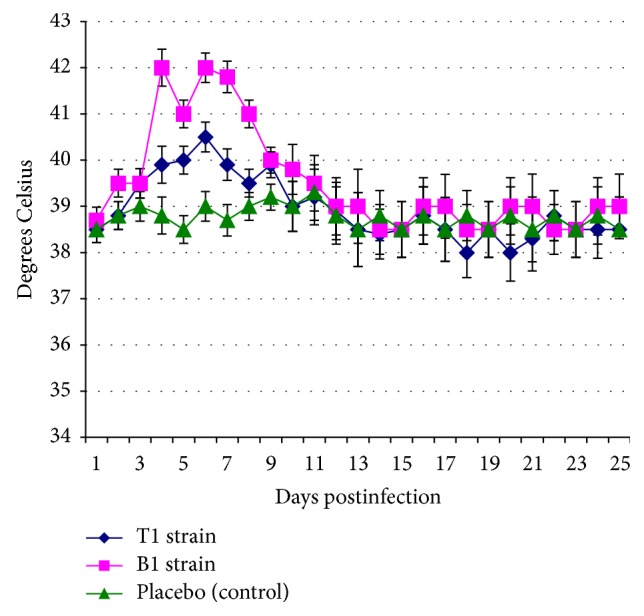
The data on the dynamics of rectal temperature in 4–6-month-old calves after aerosol challenge by bovine viral diarrhea virus cytopathic strains of subtype 1b with the dose of 6 log_10_ TCD_50_. The values are presented as *M* ± *I*_95_ (*M*, mean value; *I*_95_, 95% confidence interval) for *n* = 3, the number of animals per time point (1–10 days postinfection, p.i.); *n* = 2, the number of animals per time point (11–25 days p.i.); values obtained for both strains 4–9 days p.i. are higher than in the control according to Mann-Whitney *U* test and Student's *t*-test at *p* ≤ 0.05; values obtained for B1 strains 2 and 4–8 days p.i. are higher than for T1 strain according to Mann-Whitney *U* test and Student's *t*-test at *p* ≤ 0.05.

**Figure 4:**
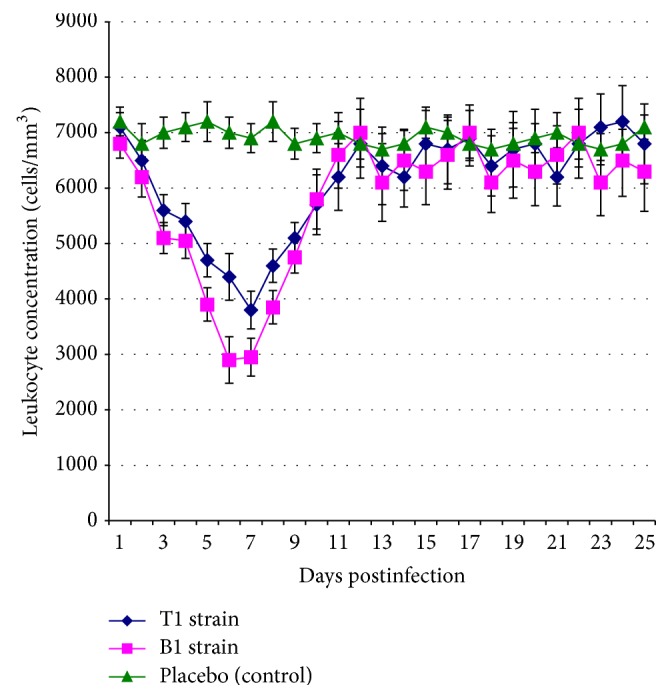
The data on the dynamics of leukocyte concentration in 4–6-month-old calves after aerosol challenge by bovine viral diarrhea virus cytopathic strains of subtype 1b with the dose of 6 log_10_ TCD_50_. The values are presented as *M* ± *I*_95_ (*M*, mean value; *I*_95_, 95% confidence interval) for *n* = 3, the number of animals per time point (1–10 days postinfection, p.i.); *n* = 2, the number of animals per time point (11–25 days p.i.); values obtained for both strains 3–10 days p.i. are less than in the control according to Mann-Whitney *U* test and Student's *t*-test at *p* ≤ 0.05; values obtained for the B1 strain 5–8 days p.i. are less than for T1 strain according to Mann-Whitney *U* test and Student's *t*-test at *p* ≤ 0.05.

It was found that both tested BVDV1 strains induced the infectious process of varying intensity and manifestations in all infected calves. B1 strain was the most virulent and induced manifestation of clinical symptoms in all the calves after 3-4 days p.i.: depression, severe hyperthermia (Figure 3), serous nasal discharge, lacrimation and catarrhal conjunctivitis, allocating viscous saliva from the oral cavity, sneezing, and coughing. On the 7th day p.i. we observed the increase of the discharge from the nasal cavities of calves. Leukopenia (Figure 4) was simultaneously recorded with the rise of body temperature in all of the animals (Figure 4). During 3 days (from day 4 to day 6 p.i.) one calf demonstrated short-term diarrhea and dark brown stool with a fetid smell. All animals recovered from 12 to 14 days p.i. The autopsy of one of the calves registered the presence of frothy fluid in the trachea and bronchi, increase in regional (lung) lymph nodes, and hyperemia mucosa of the small intestine, increasing the mesenteric lymph nodes on 10th day p.i. Genetic BVDV1 material was observed in all organs and tissues of the animals (see Table 2).

On 3rd-4th day p.i. calves challenged with BVDV1 T1 strain demonstrated depression and development of clinical signs of disease (including hyperthermia, Figure 3, and leukopenia, Figure 4), similar to those seen in animals challenged with a BVDV1 B1 strain but less pronounced. All sick animals recovered from 12 to 14 days p.i. Macroscopic examination of the calf of this group did not reveal any pathological changes.

On the 25th p.i. all of the experimental animals had antibody titers (1 : 8–1 : 16) in their blood sera. During the entire period of observation control groups of animals showed no pathological changes in all tested parameters.

## 4. Discussion

We studied the virulent properties of five strains BVDV1 belonging to three subtypes, isolated by us from the animals in the Siberian region in this paper. Three strains were isolated from the lungs of calves with respiratory pathology, one from the sperm of bull from the center of artificial insemination, and one from blood serum of persistently infected heifers. Despite the different sources of isolation from cattle of different ages and different pathological conditions, our results showed that all of noncytopathic and cytopathic BVDV1 strains of three subtypes possessed virulent properties for seronegative 4–6-month-old calves but with varying degrees of severity.

The animals were infected using the same dose of viral material (6 log_10_ TCID_50_). A titration of noncytopathic strains was performed in primary calf testis cell culture by registering the presence of viral genetic material by PCR. Possibly, the obtained values of virus titers were higher than those that could be obtained using immunoperoxidase test which detects the presence of viral antigen including live virus in the cell culture. The main external clinical signs of the disease (mild respiratory syndrome and transient diarrhea) and macroscopic changes in the respiratory and intestinal organs have been reported only in calves infected with BVDV Bison strain (subtype 1d) and BVDV B1 strain (subtype 1b), relating, respectively, to the noncytopathic and cytopathic biotypes. At the same time, all calves infected with investigated strains developed sings of depression, loss of appetite, body hyperthermia, transit leukopenia, and local spread of the virus in various organs and tissues. Genetic materials of Bison and B1 strains were detected most frequently in a wide range of the organs.

Terms revealing leukopenia in animals of all tested groups were the same and were determined after 3–5 days p.i., that is, either to the development of visible clinical reaction to the introduction of the virus, or simultaneously with it. According to the literature [13, 23], the rate of infected animals had pathogentic and prognostic value as shown by the development of their immune suppression, creating the conditions for superinfection with other pathogens of viral and bacterial origin.

In our experiments practically all calves (9 of 10) formed 25 days p.i. (observation period) a humoral immune response to BVDV1, but antibodies titers detected in serum neutralization test did not exceed 1 : 16. Only one calf infected with a BVDV1 T2 strain had no antibodies in blood serum. Relatively low levels of seroconversions in calves after recovery were connected with the immunosuppression property of BVDV1, essentially tightening the period of its occurrence (3–12 weeks p.i.) and scale [15]. Perhaps this index would be much higher if the study of antibodies to BVDV1 in calves was conducted at a later period: 2-3 months p.i. The results of our work to some extent are consistent with the data of other researchers [24], who conducted experiments on the infection of the 6 seronegative calves with laboratory BVDV1 NADL and Oregon C24V strains. These calves had clinical signs of disease (cough, serous discharge from the eyes, biphasic fever, transit leukopenia, and decreasing the lymphocyte number). The seroconversion only in five animals was observed 14 days p.i. (titers of antibodies to the virus were 1 : 2). Given the high degree of homology between the two T2 and NADL BVDV strains belonging to the same subtype (1a), in our experiments we had the right to expect the higher values of antibody titers in the calves infected with T2 strain, compared with the other strains. However, that did not happen. It can also be explained by the relatively short period of time chosen by us (25 days p.i.) between challenge and sampling for serological testing. The values of antibody titers during this period probably did not have time to reach their maximum.

The role of acute BVDV infections in respiratory disease in cattle is controversial. In part, this is due to the difficulty in differentiating acute versus persistent infection in field cases in cattle, including young calves [25]. In experimental infections with BVDV2 isolates, neither pneumonia nor immunohistochemical evidence of pulmonary infection was reported [26]. Similarly, BVDV antigen was identified only in peribronchiolar lymphoid tissue and no pulmonary lesions were noted [27]. In contrast, other authors registered gross histological lesions consisting of multifocal bronchointerstitial pneumonia (involving 10–25% of affected lungs), bone marrow hypoplasia and necrosis, and minimal erosive lesions in the alimentary tract, revealing widespread viral antigen usually within epithelial cells, smooth muscle cells, and mononuclear phagocytes in multiple organs, including lung, Peyer's patches, gastric mucosa, thymus, adrenal gland, spleen, lymph nodes, bone marrow, and skin [28]. These data support the concept that the pneumopathogenicity, and perhaps pulmonary target cell tropism, varies amongst acutely infecting BVDV isolates [29]. In connection with this, the fact of the presence of virus in lung tissue has a certain scientific interest. In our experiments with using PCR, virus was also detected in the tissue. The appearance of virus could be either due to its reproduction in the tissue or with its skid through the bloodstream in short-term viremia.

For the first time an experimental reproduction of a respiratory disease by BVDV1 was reported by Potgieter et al. in 1984 [23]. In three 6-month-old calves infected with the virus, developed signs of mild respiratory disease were in the form of fever, discharge from the nose, and rare cough. At autopsy they revealed lesions from 2 to 7% of the lung surface. It is known that the most common form of viral diarrhea is subclinical and often goes unnoticed by veterinary specialists. In this case, an important biological property of the pathogen is a transit immunosuppression, during which the animals become susceptible to the development of respiratory illnesses due to the enhanced reproduction of coinfectious microorganisms.

Role of noncytopathic BVDV biotypes in the occurrence of the respiratory diseases for a long time was underestimated and the ability of this biotype strains to induce clinical respiratory disease in cattle has been discussed since the beginning of the 90s of the last century [3]. Thus, in this period of time in the United States and Canada several noncytopathic BVDV strains belonging to type 2, causing thrombocytopenia, hemorrhages, leukopenia, fever, diarrhea, and loss of animals during primary acute infection [30] have been allocated. This fact stimulated interest in the study of the pathogenic properties of the virus. However, most noncytopathic BVDV2 strains can cause either subclinical or “moderate” acute form of the disease [31, 32], which is also confirmed by the results of our experiments.

Knowledge about the role of the BVDV1 subtypes in the etiology of respiratory diseases of young cattle is limited. It is believed that the etiology of the respiratory diseases often involves subtype 1b isolates [32]. Experiments by Galav et al. [33] demonstrated the pathogenicity of the Indian virus strain of subtype 1b for 7–9-month-old calves with p.i. developed symptoms of respiratory disease, the two-phase temperature reaction, mild diarrhea, mild leukopenia, and thrombocytopenia. Viremia occurred from 3 to 15 days p.i., and seroconversion was detected on 15 days p.i. The animals demonstrated interstitial pneumonia, easy gastroenteritis, and signs of systemic spread of the virus. But our experiments with not only the BVDV strains of subtypes 1b, but also 1d, showed in calves the main disease signs, similar to the above, and confirmed the results obtained by other researchers [34], who registered a primary respiratory disease in colostrum deprived calves experimentally infected with subtype 1d strain.

Inoculation of cytopathic BVDV subtype 1a strain, isolated from cattle in China, in experimental infected calves resulted in depression, cough, fever above 40°C, and a reduction in the number of white blood cells by 40%, which the authors described as a moderate degree of pathogenicity of this strain [35]. The results of our research in this field, but using the cytopathic BVDV strain belonging to other subtype (1b), are generally consistent with the data given.

Currently, there are at least 16 subtypes of BVDV1, but in the available scientific literature there is not enough data on their virulent properties for calves. It is known that the clinical signs of BVDV infection range from asymptomatic through mild transient sign to severe acute disease, including fever, diarrhea, anorexia, respiratory disorder, reproductive failure, increased susceptibility to secondary infections due to the immunosuppression, congenital abnormalities, and hemorrhage syndrome accompanied by a high mortality rate [30, 36]. Noncytopathic BVDV1 strains cause subclinical disease, and signs of acute infection depend on the viral strain and immune status of the animal. The mechanism of BVDV-induced leukopenia and thrombocytopenia has not been completely characterized; however, these hematological abnormalities might be involved in disease outcomes of BVDV infection.

The differences in pathogenicity between the types/subtypes of the virus are associated with mutations caused by errors of the RNA-dependent RNA-polymerase and recombinations. Because of the frequent mutations during replication of RNA, virus exists as distinct, but closely related mutants (“quasispecies”) undergoing continuous selection. In this regard, the strain pathogenicity varies considerably and has a “strain” dependence [36, 37]. The main difference between the cytopathic and noncytopathic biotypes of virus at the molecular level is an expression of nonstructural polypeptide NS3. Noncytopathic virus expresses the single polypeptide with a size of 125 KD (NS2-3 or p125), and cytopathic isolates have two proteins NS2-3 and NS3 [38, 39].

As already mentioned, the size of Russian dairy farms is large enough, and the arrival of animals from different sources creates the conditions for BVDV circulation among susceptible gender and age groups of animals. Therefore, when selecting strains of BVDV, we relied on published data on the etiologic role of subtypes 1a, 1b, and 1d in the occurrence of respiratory, gynecological, and other pathology [40–42]. In addition, the relevance of the our research with the BVDV subtypes 1a and 1b was due to the fact that the subtypes are used in the production of appropriate vaccines that are employed all over the world, including Russia [42].

## 5. Conclusions

This paper presents the results relating to the study of the virulent properties of five BVDV1 strains, first isolated and typed in Siberia region. The results showed that all investigated cytopathic and noncytopathic BVDV1 strains, belonging to different subtypes (1a, 1b, and 1d) and circulating in Siberia, caused the development of infection in seronegative calves after aerosol challenge with the dose of 6 log_10_ TCID_50_. In all cases, the animals recovered from 12 to 15 days p.i. and in most cases they formed a low level humoral immune response 25 days p.i. Our results confirmed the presence of virulent properties of five BVDV1 strains and showed the need for further research on the molecular epidemiology of the disease, development of more effective diagnostic systems, and optimization of regional disease control programs with use of vaccines. These activities may allow reducing the risk of infection in dairy cattle industry, initiated by other pathogenic viruses and bacteria, significantly. This is the first Russian work, which was performed to study virulent properties of the Siberian BVDV1 strains of three different subtypes belonging to two biotypes in seronegative calves.

## Figures and Tables

**Table 1:** Pathogenic properties of bovine viral diarrhea virus (BVDV) noncytopathic strains of type 1 after aerosol challenge of 4–6-month-old calves with the dose of 6 log_10_ TCD_50_.

Name of the strain/isolation source	Subtype	Manifestation form of the disease	Time of leukopenia (days postinfection), the lowest concentration of leukocytes (cells/mm^3^, *M* ± *I*_95_ for *n* = 3)	Presence of antibodies to BVDV	Type of organ system with BVDV genome
Bison/calf lung	1d	Acute (acute respiratory infection, diarrhea)	4–9, 2700 ± 300^*∗*^	+	Lymphoid (spleen, pulmonary lymph nodes), respiratory (lungs), and intestinal (small intestine, duodenum, ileum, and mesenteric lymph nodes)

T2/bull sperm	1a	Subclinical	4–8, 3500 ± 300	±	Lymphoid and respiratory (spleen, pulmonary lymph nodes)

Bor/heifer blood serum	1b	Subclinical	3–9, 3700 ± 300	+	Lymphoid (spleen, mesenteric lymph nodes, Peyer's patches)

*M*, mean value.

*I*_95_, 95% confidence interval.

*n*, number of animals.

^*∗*^The value is below those for T2 and Bor strains according to the Mann-Whitney *U* test and Student's *t*-test at *p* ≤ 0.05.

**Table 2:** Pathogenic properties of bovine viral diarrhea virus (BVDV) cytopathic strains of type 1 after aerosol challenge of 4–6-month-old calves with the dose of 6 log_10_ TCD_50_.

Name of the strain/isolation source	Subtype	Manifestation form of the disease	Time of leukopenia (days postinfection), the lowest concentration of leukocytes (cells/mm^3^, *M* ± *I*_95_ for *n* = 3)	Presence of antibodies to BVDV	Type of organ system with BVDV genome
B1/calf lung	1b	Acute (acute respiratory infection, diarrhea)	3–10, 2800 ± 400^*∗*^	+	Lymphoid (spleen, pulmonary lymph nodes), respiratory (lungs), and intestinal (small intestine, duodenum, ileum, and mesenteric lymph nodes)

T1/calf lung	1b	Acute (acute respiratory infection, diarrhea)	4–9, 3800 ± 400	±	Lymphoid (spleen, mesenteric lymph nodes, Peyer's patches, and mesenteric lymph nodes)

*M*, mean value.

*I*_95_, 95% confidence interval.

*n*, number of animals.

^*∗*^The value is below that for T1 strain according to the Mann-Whitney *U* test and Student's *t*-test at *p* ≤ 0.05.

## References

[1] Gulyukin M. I., Yurov K. P., Glotov A. G., Donchenko N. A. (2013). Bovine viral diarrhea control in Russian Federation. *Voprosy Virusologii*.

[2] Ridpath J. F. (2010). Bovine viral diarrhea virus: global status. *Veterinary Clinics of North America-Food Animal Practice*.

[3] Deregt D., Loewen K. G. (1995). Bovine viral diarrhea virus: biotypes and disease. *Canadian Veterinary Journal*.

[4] Lindberg A. L. E. (2003). Bovine viral diarrhoea virus infections and its control. A review. *Veterinary Quarterly*.

[5] Ridpath J. F., Bolin S. R., Dubovi E. J. (1994). Segregation of Bovine viral diarrhea virus into genotypes. *Virology*.

[6] Vilček Š., Paton D. J., Durkovic B. (2001). Bovine viral diarrhoea virus genotype 1 can be separated into at least eleven genetic groups. *Archives of Virology*.

[7] Vilček Š., Ďurkovič B., Kolesárová M., Greiser-Wilke I., Paton D. (2004). Genetic diversity of international bovine viral diarrhoea virus (BVDV) isolates: identification of a new BVDV-1 genetic group. *Veterinary Research*.

[8] Giangaspero M., Apicella C., Harasawa R. (2013). Numerical taxonomy of the genus Pestivirus: new software for genotyping based on the palindromic nucleotide substitutions method. *Journal of Virological Methods*.

[9] Giangaspero M., Harasawa R. (2014). Characterization of genotypes among bovine viral diarrhea virus type 1 strains according to palindromic nucleotide substitutions in the genomic 5′-untranslated region. *Journal of Virological Methods*.

[10] Glotov A. G., Glotova T. I., Yuzhakov A. G., Zaberezhniy A. D., Aliper T. I. (2009). Isolation of noncytopathogenic genotype 2 bovine viral diarrhea virus from the cattle mucosa in the Russian Federation. *Voprosy Virusologii*.

[11] Kelling C. L., Steffen D. J., Topliff C. L., Eskridge K. M., Donis R. O., Higuchi D. S. (2002). Comparative virulence of isolates of bovine viral diarrhea virus type II in experimentally inoculated six- to nine-month-old calves. *American Journal of Veterinary Research*.

[12] Seong G., Oem J.-K., Choi K.-S. (2013). Pathogenetic differences after experimental infection of calves with Korean non-cytopathic BVDV-1 and BVDV-2 isolates. *Veterinary Immunology and Immunopathology*.

[13] Molina V., Risalde M. A., Sánchez-Cordón P. J. (2014). Cell-mediated immune response during experimental acute infection with bovine viral diarrhoea virus: evaluation of blood parameters. *Transboundary and Emerging Diseases*.

[14] Raya A. I., Gomez-Villamandos J. C., Bautista M. J. (2015). Role of thymic epithelial cells in lymphoid depletion after experimental infection with the noncytopathogenic BVDV1 strain 7443. *Veterinary Pathology*.

[15] Goyal S. M., Ridpath J. F. (2005). *Bovine Viral Diarrhea Virus: Diagnosis, Management, and Control*.

[16] Pedrera M., Gómez-Villamandos J. C., Molina V., Risalde M. A., Rodríguez-Sánchez B., Sánchez-Cordón P. J. (2012). Quantification and determination of spread mechanisms of bovine viral diarrhoea virus in blood and tissues from colostrum-deprived calves during an experimental acute infection induced by a non-cytopathic genotype 1 strain. *Transboundary and Emerging Diseases*.

[17] Palomares R. A., Sakamoto K., Walz H. L., Brock K. V., Hurley D. J. (2015). Acute infection with bovine viral diarrhea virus of low or high virulence leads to depletion and redistribution of WC1^+^
*γδ* T cells in lymphoid tissues of beef calves. *Veterinary Immunology and Immunopathology*.

[18] Glotov A. G., Glotova T. I., Ryabchikova Y. I., Sergeev A. N. (2006). Isolation and characterization of bovine diarrhea viral isolates. *Voprosy Virusologii*.

[19] OIE (2015). Bovine viral diarrhea. *Manual of Diagnostic Tests and Vaccines for Terrestrial Animals*.

[20] Ridpath J. F., Bolin S. R. (1998). Differentiation of types 1a, 1b and 2 bovine viral diarrhea virus (BVDV) by PCR. *Molecular and Cellular Probes*.

[21] Jain N. C. (1986). *Schalm's Veterinary Hematology*.

[22] Zaks L. *Statistical Estimation*.

[23] Potgieter L. N., McCracken M. D., Hopkins F. M., Walker R. D., Guy J. S. (1984). Experimental production of bovine respiratory tract disease with bovine viral diarrhea virus. *American Journal of Veterinary Research*.

[24] Polak M. P., Zmudzinski J. F. (2000). Experimental inoculation of calves with laboratory strains of bovine viral diarrhea virus. *Comparative Immunology, Microbiology and Infectious Diseases*.

[25] Richer L., Marois P., Lamontagne L. (1988). Association of bovine viral diarrhea virus with multiple viral infections in bovine respiratory disease outbreaks. *The Canadian Veterinary Journal*.

[26] Marshall D. J., Moxley R. A., Kelling C. L. (1996). Distribution of virus and viral antigen in specific pathogen-free calves following inoculation with noncytopathic bovine viral diarrhea virus. *Veterinary Pathology*.

[27] Wilhelmsen C. L., Bolin S. R., Ridpath J. F., Cheville N. F., Kluge J. P. (1990). Experimental primary postnatal bovine viral diarrhea viral infections in six-month-old calves. *Veterinary Pathology*.

[28] Ellis J. A., West K. H., Cortese V. S. (1998). Lesions and distribution of viral antigen following an experimental infection of young seronegative calves with virulent bovine virus diarrhea virus-type II. *Canadian Journal of Veterinary Research*.

[29] Potgieter L. N., McCracken M. D., Hopkins F. M., Guy J. S. (1985). Comparison of the pneumopathogenicity of two strains of bovine viral diarrhea virus. *American Journal of Veterinary Research*.

[30] Carman S., van Dreumel T., Ridpath J. (1998). Severe acute bovine viral diarrhea in Ontario, 1993–1995. *Journal of Veterinary Diagnostic Investigation*.

[31] Spagnuolo-Weaver M., Allan G. M., Kennedy S., Foster J. C., Adair B. M. (1997). Distribution of cytopathic and noncytopathic bovine viral diarrhea virus antigens in tissues of calves following acute experimental infection. *Journal of Veterinary Diagnostic Investigation*.

[32] Fulton R. W., Saliki J. T., Confer A. W. (2000). Bovine viral diarrhea virus cytopathic and noncytopathic biotypes and type 1 and 2 genotypes in diagnostic laboratory accessions: clinical and necropsy samples from cattle. *Journal of Veterinary Diagnostic Investigation*.

[33] Galav V., Mishra N., Dubey R. (2007). Pathogenicity of an Indian isolate of bovine viral diarrhea virus 1b in experimentally infected calves. *Research in Veterinary Science*.

[34] Baule C., Kulcsár G., Belák K. (2001). Pathogenesis of primary respiratory disease induced by isolates from a new genetic cluster of bovine viral diarrhea virus type I. *Journal of Clinical Microbiology*.

[35] Wang W., Shi X., Tong Q. (2014). A bovine viral diarrhea virus type 1a strain in China: isolation, identification, and experimental infection in calves. *Virology Journal*.

[36] Ridpath J. F., Bolin S. R. (1995). The genomic sequence of a virulent bovine viral diarrhea virus (BVDV) from the type 2 genotype: detection of a large genomic insertion in a noncytopathic BVDV. *Virology*.

[37] Ridpath J. F., Fulton R. W., Kirkland P. D., Neill J. D. (2010). Prevalence and antigenic differences observed between bovine viral diarrhea virus subgenotypes isolated from cattle in Australia and feedlots in the southwestern united states. *Journal of Veterinary Diagnostic Investigation*.

[38] Ridpath J., Goyal S. M., Ridpath J. F. (2005). Classification and molecular biology. *Bovine Viral Diarrhea Virus: Diagnosis, Management and Control*.

[39] Bolin S. R., Grooms D. L. (2004). Origination and consequences of bovine viral diarrhea virus diversity. *Veterinary Clinics of North America-Food Animal Practice*.

[40] Fulton R. W., Ridpath J. F., Saliki J. T. (2002). Bovine viral diarrhea virus (BVDV) 1b: predominant BVDV subtype in calves with respiratory disease. *Canadian Journal of Veterinary Research*.

[41] Evermann J. F., Ridpath J. F. (2002). Clinical and epidemiologic observations of bovine viral diarrhea virus in the northwestern United States. *Veterinary Microbiology*.

[42] Fulton R. W. (2015). Impact of species and subgenotypes of bovine viral diarrhea virus on control by vaccination. *Animal Health Research Reviews*.

